# Influence of ZrO_2_ Nanoparticle Addition on the Optical Properties of Denture Base Materials Fabricated Using Additive Technologies

**DOI:** 10.3390/nano12234190

**Published:** 2022-11-25

**Authors:** Abdulrahman Khattar, Majed H. Alsaif, Jawad A. Alghafli, Ali A. Alshaikh, Ali M. Alsalem, Ibrahim A. Almindil, Abdulsalam M. Alsalman, Ali J. Alboori, Abdullah M. Al-Ajwad, Hussain M Almuhanna, Soban Q. Khan, Hamad S. AlRumaih, Mohammed M. Gad

**Affiliations:** 1 College of Dentistry, Imam Abdulrahman Bin Faisal University, P.O. Box 1982, Dammam 31441, Saudi Arabia; 2 Department of Dental Education, College of Dentistry, Imam Abdulrahman Bin Faisal University, P.O. Box 1982, Dammam 31411, Saudi Arabia; 3 Department of Substitutive Dental Sciences, College of Dentistry, Imam Abdulrahman Bin Faisal University, P.O. Box 1982, Dammam 31441, Saudi Arabia

**Keywords:** 3D printing, ZrO_2_ nanoparticles, denture base, translucency

## Abstract

This study investigated the translucency of 3D-printed denture base resins modified with zirconium dioxide nanoparticles (ZrO_2_NPs) under thermal cycling. A total of 110 specimens were fabricated and divided into 3 groups according to the materials, i.e., heat-polymerized resin, and 3D-printed resins (NextDent, and ASIGA). The 3D-printed resins were modified with 0, 0.5, 1, 3, and 5 wt.% of ZrO_2_NPs. All the specimens were subjected to 5000 thermal cycles. The translucency was measured using a spectrophotometer. The results showed that the heat-polymerized resin had considerably higher translucency than the 3D-printed resins. Compared to the unmodified group, the translucency decreased significantly after adding 5% ZrO_2_NPs to NextDent and 3% ZrO_2_NPs to ASIGA resins. The highest translucency was achieved for NextDent by adding 0.5% ZrO_2_NPs and for ASIGA without any ZrO_2_NPs. It was found that the average concentration level in ASIGA was significantly higher than that in NextDent. These findings revealed that 3D-printed resins have lower translucency than heat-polymerized acrylic resin, and adding ZrO_2_NPs at low concentrations did not affect the translucency of the 3D-printed resins. Therefore, in terms of translucency, 3D-printed nanocomposite denture base resins could be considered for clinical applications when ZrO_2_NPs are added at low concentrations.

## 1. Introduction

The geriatric population is growing rapidly worldwide as healthcare advancements increase life expectancy [1]. One of the most common oral conditions that affect old people is complete edentulism [2,3]. Despite the availability of treatment modalities, a complete denture is the most popular treatment for edentulism [4,5]. Poly(methyl methacrylate) (PMMA) is commonly used for the fabrication of removable dental prostheses owing to its good biocompatibility, low cost, and easy fabrication and repair [6]. However, PMMA tends to adsorb water, compromising its physical properties [7]. Moreover, it has poor surface characteristics, leading to denture stomatitis because of easy *Candida albicans* (*C. albicans*) adhesion and biofilm formation [6]. Some techniques have been recently developed to overcome these limitations, such as denture base coating, loading antifungal drugs, and incorporating nanoparticles (NPs) to improve the mechanical properties [6].

Denture esthetics is defined as the beauty and attractiveness of a person based on the effect produced by the prosthesis [8]. Owing to better awareness, the number of patients demanding esthetics has risen dramatically [8]. There is strong evidence that the success of complete dentures is closely related to the acceptance of their esthetics [9,10]. The esthetics of denture-base acrylic resins are significantly influenced by the translucency of removable prostheses [11].

The light traveling through a material experiences transmission, absorption, reflection, and scattering interactions with other light sources [12]. A translucent material allows some light to pass through it, and the objects on the other side of the translucent material appear fuzzy and unclear [13,14]. The translucency of a denture base gives a natural look due to the “chameleon effect” by allowing the surroundings to be reflected and viewed through it [15]. The color and appearance of the underlying soft tissue are crucial esthetic requirements for an acrylic denture base [15]. PMMA is a highly versatile resin for incorporating fillers to achieve translucency [15].

Different NPs have been used in PMMA denture base materials [16,17,18,19]. Zirconium dioxide (ZrO_2_), aluminum dioxide (Al_2_O_3_), titanium dioxide (TiO_2_), silver nanoparticles (AgNPs), and silicon dioxide (SiO_2_) can reinforce denture bases and have different effects on the final denture properties [20]. The properties of the added NPs, including their shape, size, concentration, and interaction with the matrix, define the final characteristics of the nanocomposite [21]. The NPs have a nanoscale size and large specific surface area relative to their volume [22,23,24]. These unique properties allow strong interfacial interaction with the organic polymers, resulting in a nanocomposite with novel mechanical, chemical, and optical properties [23].

Aszrin et al. (2016), evaluated different types of NPs incorporated in PMMA and found that the translucency of PMMA exhibited unpredictable negative results at different NP concentrations [25]. Likewise, Gad et al. (2018), and Lee et al. (2007) revealed that the decrease in translucency was directly related to the concentration of the added NPs [15,26]. In addition, introducing metal oxide NPs may change the translucency of the matrix material owing to the natural color of the NPs. However, ZrO_2_ NPs are white and, thus, are less likely to affect the color of the resin [19,27].

Nowadays, removable dentures can be manufactured using digital processes such as computer-aided design and computer-aided manufacturing (CAD-CAM), which have been widely used in dentistry. The fabrication process of digital dentures was first established as a subtractive technique in which the dentures were manufactured from prefabricated resin blocks. Recently, additive manufacturing, also known as 3D printing, was introduced [28,29] to build objects via a layer-by-layer process [28,29]. 3D printing technology can eliminate the need for conventional molds and tools and simplify the fabrication process of a complete denture [28,29,30]. In addition, 3D printing technology uses a concentrated selective laser beam to melt filament locally and can reduce the consumption of material in the polymerization process [31]. Furthermore, 3D printing reduces the procedure time and laboratory work [32], providing a significant improvement in terms of tissue adaptation and duplication of existing dentures in use [33]. Moreover, by using 3D-printing method, the margin of error made by laboratory technicians can be minimized, offering higher accuracy than that of conventional methods [33,34,35,36].

Several studies have examined the effects of additives on 3D-printed materials [37,38,39,40]. Chen et al. (2018) discovered that a 3D-printed resin containing cellulose nanocrystals and AgNPs (0–0.1 wt.%) exhibited higher flexural and impact strengths [37]. Mubarak et al. (2020) found that the tensile strength, tensile modulus, and flexural strength of 3D-printed materials increased with the addition of less than 1 wt.% silver-titanium dioxide NPs [38]. Aati et al. (2021) reported that a 3D-printed resin modified with ZrO_2_ showed long-term improvement in provisional restorations [39]. Moreover, the hardness and flexural and impact strengths of a 3D-printed resin were enhanced when SiO_2_NPs were incorporated [40].

A previous article published by Gad et al. (2022) [40] demonstrated that after adding NPs to 3D-printed PMMA resin, most of the properties of the PMMA were significantly enhanced, except for surface hardness and roughness. However, its optical properties have not yet been assessed [39]. Evaluating the optical properties of a resin material is important to ensure good esthetic results. To the best of our knowledge, no prior studies have examined the effect of adding ZrO_2_NPs on the translucency of 3D-printed denture base resins. Consequently, this in vitro study investigated the translucency of 3D-printed denture base resins modified with ZrO_2_NPs through thermal cycling experiments. The null hypothesis is that adding ZrO_2_NPs to the 3D-printed resin does not affect the translucency of the nanocomposite.

## 2. Materials and Methods

Power analysis was used to count the in vitro samples. According to the World Health Organization formulae, a study power of 80%, a significance level of 5%, and a marginal error of 5% were determined. A total of 110 specimens were divided into 11 groups: five groups of two different 3D-printed resins (NextDent and ASIGA) and one heat polymerized resin.

### 2.1. Preparation of Nanocomposite Mixture

NextDent and ASIGA were employed in this study along with heat-polymerized PMMA. ZrO_2_NPs (99.9% purity, Sigma-Aldrich, St. Louis, MO, USA) were added to the 3D-printed resins at different concentrations (0, 0.5, 1, 3, and 5 wt.%) [41]. Based on earlier SEM and TEM analyses [15,41,42,43,44], the average granularity and surface area of the ZrO_2_NPs were 40 nm and 9 m^2^/g, respectively [45]. To enhance the bonding between the ZrO_2_NPs and resin matrix, a silane coupling agent 3- (trimethoxysilyl) propyl methacrylate (Shanghai Richem International Co., Ltd., Shanghai, China) was used to treat the surface of ZrO_2_NPs by creating reactive groups through the silanization process. The silane coupling agent was dissolved in acetone and then ZrO_2_NPs were added to the mixture followed by stirring for 60 min. Then, a rotary evaporator was used for acetone eliminations followed by cooling to obtain the silanized ZrO_2_NPs. The silanized ZrO_2_NPs were added to the 3D-printing resins at various concentrations. Following previous studies, the modified liquid resins were thoroughly mixed and stirred for 30 min [17,27].

#### 2.1.1. Preparation of Heat-Polymerized Acrylic Resin Specimens

Heat-polymerized acrylic resin specimens (Major.Base.20 MAJOR, Prodotti Dentari S.p.A. moncalieri, Italy), were manufactured based on a conventional method for denture processing [6] and used as a control. A metal mold (15 mm × 2 mm) was used to fabricate wax specimens, which were invested in dental stone followed by wax removal to crate mold spaces for acrylic resin packing at the dough stage. After packing, the flask was placed into a thermal polymerization unit to complete the polymerization cycle (heated to 73 °C for 90 min and then heated to 100 °C for an additional 30 min) [6].

#### 2.1.2. Preparation of 3D-Printed Specimens

An open-source CAD system (123D design, Autodesk, version 2.2.14, San Rafael, CA, USA) was used to design the 3D-printed specimens. The files were saved as STL files and printed using a 3D-printing machine with the previously mentioned dimensions. A pure resin was mixed using anLC 3D Mixer (NextDent, Soesterberg, The Netherlands) for 120 min. After mixing, specific concentrations of ZrO_2_NPs were added to the resin mix and distributed into several bottles. These bottles were then shaken using the same mixer for 30 min before printing. The printing details for each layer are listed in Table 1 [46], along with details regarding the printing and post-printing processes, such as the intensity of ultraviolet (UV) light, rinsing and cleaning materials, and post-curing machines, and time. Low-speed rotary tools were used to remove the excess resin. Finishing and polishing were performed using a polishing cloth and polishing machine under wet conditions. The specifications of the tools, materials, and machines are listed in Table 2 [47].

### 2.2. NP Distribution and Bonding Analysis

The complete and even of distribution of NPs within the resins was analyzed using scanning electron microscopy (SEM, FEI, Inspect S50, Brno, Czech Republic at 20 kV). Fourier transform infrared spectroscopy (FTIR) (Nicolet 6700, FTIR spectrometer, Thermo Fisher Scientific, Waltham, United States) was used to explore the bonding of the specimens prepared with various ZrO_2_NP concentrations (0 wt, 0.5 wt, 1 wt, 3 wt, and 5 wt.%). To obtain the FTIR spectra, the specimens were scanned between 4000 and 400 cm^−1^. The specimen preparations steps for SEM and FTIR analyses were detailed in our previous study [41].

### 2.3. Thermal Cycling Procedures

Before the specimens were subjected to thermocycling, they were rinsed with water, followed by coarse and fine rubber tips. A thermocycling machine was used to simulate intraoral temperature changes over six months. The number of cycles, temperature, dwell time, and machine manufacturers are listed in Table 1 [48,49].

### 2.4. Translucency Test

Reflectance values were determined using a spectrophotometer (Color-Eye^®^ 7000 A, X-Rite, Carlstadt, NJ, USA). A small-aperture viewing area (10 mm × 7.5 mm) was selected. A white tile and black trap were used to calibrate the spectrophotometer following the manufacturer’s recommendations. Every specimen was stabilized against the port, supported at the back with the black or white reference material and then the support arm was closed. For every disc, color measurements were performed against each background using the (L*, a*, b*) coordinates defined by the Commission Internationale de l’Eclairage (CIE) system. An average of three readings was obtained for each specimen using the spectrophotometer software. The data were tabulated and the translucency (TR) was calculated using the following equation: TR = [(L*white − L*black)^2^ + (a*white − a*black)^2^ + (b*white − b*black)^2^] ^1/2^ [25,50].

### 2.5. Statistical Analysis

A statistical package for the social sciences (SPSS Statistics for Windows, Version 27.0. Armonk, NY: IBM Corp) was used for data entry and analysis. In the descriptive data analysis, the means and standard deviations were computed. The normality of the data was tested using the Shapiro–Wilk test, and insignificant results indicated that the data were normally distributed. Hence, parametric tests were employed for inferential analysis. One-way analysis of variance (ANOVA) was used to study the variation in the tested properties at different ZrO_2_NP concentration levels. In addition, two-way ANOVA was used to study the combined effects of material type and concentration. Statistical significance was set as 0.05.

## 3. Results

The FTIR spectra of HP and 3D-printed resins (NextDent and ASIGA) showed some variations which suggests that the chemical structures of NextDent and ASIGA resins are different. The spectra of 3D-printed resins (NextDent and ASIGA) comparison spectra displayed similar IR bands even with the addition of ZrO_2_NP, suggesting that the 3D-printed materials modified with NPs had a uniform distribution. By comparing the spectra, it is clear that the bands of 3D-printed resins (NextDent and ASIGA) are different from those measured for HP, particularly in the spectral region between 1600 and 400 cm^−1^, highlighting the varied bonding features of the 3D-printed resins [41].

**Figure 1:**
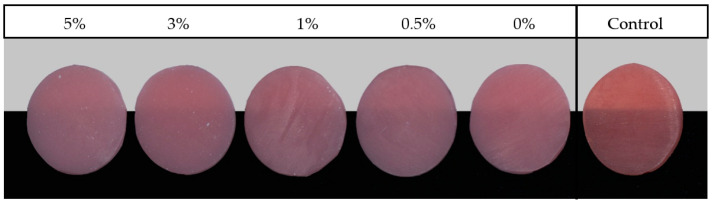
Representative photographs of the NextDent resin specimens ordered according to ZrO_2_NP concentration.

**Figure 2:**
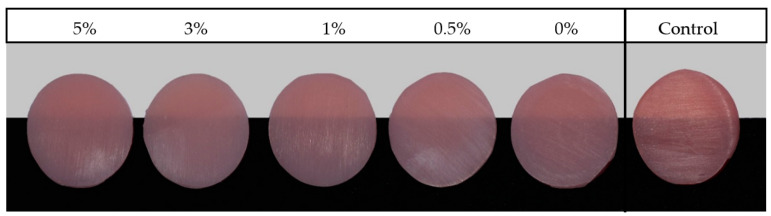
Representative photographs of the ASIGA resin specimens ordered according to ZrO_2_NP concentration.

Figure 1 and Figure 2 show photographs of the PMMA specimens with different ZrO_2_NP concentrations from the NextDent and ASIGA groups, respectively. The mean and standard deviation of the translucency are summarized in Table 3 and Table 4. The mean translucency of the control group was significantly higher than those of the NextDent and ASIGA groups (Table 3 and Table 4).

One-way ANOVA revealed significant differences in the translucency of the NextDent group (*p* < 0.001). The highest translucency was recorded for NextDent (0.5%) (6.40 ± 0.55) and the lowest for NextDent (5%) (4.91 ± 0.35). Tukey’s post hoc tests showed significantly lower translucency of the NextDent group compared with that of the control group (*p* < 0.001). The NextDent modified with 5% ZrO_2_NP showed a significant decrease in translucency compared to the unmodified NextDent. However, NextDent modified with 0.5% ZrO_2_NP showed an insignificant increase in translucency compared to the unmodified NextDent. Within the modified NextDent group, significant differences existed between 0.5% vs. 5% and 1% vs. 5%.

One-way ANOVA revealed significant differences in the translucency of the ASIGA group (*p* < 0.001) (Table 4). The highest translucency was recorded for ASIGA (0%) (9.26 ± 0.48) and the lowest for ASIGA (3%) (7.13 ± 0.47). Tukey’s post hoc tests showed significantly lower translucency of the ASIGA groups compared with that of the control group (*p* < 0.001). The ASIGA modified with 3% and 5% ZrO_2_NP exhibited a significant decrease in translucency compared with unmodified ASIGA. A significant variation was observed between the ZrO_2_NP-modified ASIGAs. Significant differences were found between 0.5% vs. 3% and 5%, and 1% vs. 3% and 5% in the ASIGA groups.

The different concentrations of NPs resulted in different mean values for the two 3D-printed resins. Therefore, a one-way ANOVA test was used, and a significant *p*-value was found between the groups (*p* < 0.001). Significant results from the ANOVA suggested the application of a post hoc test. Table 5 presents a pairwise comparison of the samples with different NP concentrations between the two materials. It was found that the average concentration of translucency in the ASIGA group was significantly higher than that in the NextDent group.

Table 6 lists the 2-way ANOVA results, where single factors (material type and NP concentration) had a significant effect on the tested property (*p* < 0.001). Moreover, it was found that the combined effect of the material and NP concentration showed a significant impact on the *p*-value (*p* = 0.003).

## 4. Discussion

This study investigated the translucency of 3D-printed denture base resins modified with ZrO_2_NPs using a thermal cycling experiment. The null hypothesis of this study assumes that adding ZrO_2_NPs to the 3D-printed resin does not affect the translucency of the nanocomposite. The null hypothesis was rejected because of a substantial difference in translucency values between the 3D-printed resins, conventional heat-polymerized resin, and ZrO_2_NP-modified 3D-printed resins. Both ASIGA and NextDent groups exhibited significant differences in translucency; however, only ASIGA yielded statistically significant results.

The oral cavity undergoes thermal stresses due to the uptake of cold and hot liquids. In this study, the specimens were subjected to 5000 thermal cycles to mimic half a year of clinical use of the prosthesis under changes in the oral cavity environment [48]. ZrO_2_ is a biocompatible metal oxide with superior surface hardness, strength, and fracture toughness [51,52]. ZrO_2_NPs have been demonstrated as a suitable reinforcing material for 3D-printed PMMA [53]. Furthermore, ZrO_2_ has thermal stability, corrosion resistance, and antibacterial and antifungal effects on *Aspergillus niger* and *C. albicans* [39,54,55]. Reinforcing PMMA with ZrO_2_NPs can endure denture base resins with the favorable characteristics of ZrO_2_ [18].

The acceptance of dental prosthetics and patient satisfaction are now dependent on meeting the esthetic requirements demanded by the patients [9]. To test the translucency of the resin materials, the samples were placed over a white background. Moreover, to evaluate the uniformity of the thickness, the samples were placed over a black background [55]. The translucency obtained from diffuse reflectance spectra measured using a UV-VIS spectrophotometer are proportional to the reflected intensity of UV light. The translucency increases to higher readings owing to the higher UV reflectance, whereas the average total translucency decreases to lower values owing to the lower reflectance [24]. The material became entirely opaque when the measured translucency was zero. Translucency also increases as the translucency readings increases [12].

Kelly et al. (1996) suggested that translucency is a key feature in material selection and a major esthetic consideration for dental prostheses [56,57]. The success of a removable prosthesis depends mainly on how translucent the denture base appears in comparison with the patient’s oral mucosa [13]. The prosthesis must have an appropriate level of translucency to appear natural. The goal is to achieve visual harmony between the removable prosthesis and underlying mucosa by giving the PMMA denture base sufficient translucency to allow the underlying soft tissues to show through, thereby achieving a “chameleon” [15].

In this study, both 3D-printed resin groups showed low translucency values compared to heat polymerized PMMA. The low translucency values of the 3D-printed resins are related to the layer-by-layer printing process, where photopolymerization occurs per printed layer [58]. Air can be trapped between the printed layers, resulting in voids that increase the levels of water sorption of 3D-printed resins. This absorbed water disrupts the UV beam, leading to low translucency [9]. Moreover, in terms of the monomer conversion rate, the polymerization technique can also explain the low translucency [59]. The low degree of polymerization of 3D-printed resins leaves unreacted monomers. When the monomer leaches out of the resin, water diffusion into the resin can occur [9]. The Fillers used in 3D-printed resins also have different refractive indices that can alter the optical properties of the composite [11,60]. It has been shown that the translucency decreases with an increasing the amount of filler added to the resin [26].

According to our findings, adding ZrO_2_NPs reduced the translucency of 3D-printed resins. Similarly, Gad et al. showed that adding ZrO_2_NPs to PMMA decreased its translucency [15]. Moreover, Aszrin et al. (2016) reported an unpredictable negative influence on translucency by introducing various concentrations of ZrO_2_, Al_2_O_3_, or SiO_2_ filler [25]. The decrease in translucency results from the optical characteristics of ZrO_2_NPs and their distribution within the resin matrix. The translucency decreases because of the crystalline structure and high opacity of ZrO_2_NPs that limit the transmission of light through the composite [15]. However, the esthetics are not affected by the white ZrO_2_NPs, unlike metal nanoparticles, such as aluminum, copper, or silver NPs [61]. ZrO_2_NPs form clusters that prohibit light transmission, thereby decreasing translucency. This is consistent with the findings of a previous study, which concluded that the agglomeration of particles within the matrix can result in the diffuse reflectance of the UV beam, which decreases translucency [62].

Translucency is affected by several factors, such as water adsorption, NP concentration, and the nature of the NPs [9]. However, in our study, all the specimens were subjected to the same treatment. Comparing the results in Table 3, the ASIGA group was more translucent than the NextDent group. This is due to the different composition of the materials and the addition of various amounts of filler. Increasing the filler content decreased the translucency, consistent with the findings of a previous report [26].

3D printing technology offers advantages for denture base resin fabrication, and the addition of NPs as a reinforcing agent is recommended to prepare nanocomposites with better properties than the original materials. A balance between esthetics and mechanical properties is required. Thus, when selecting a filler concentration that will enhance the esthetics, care must be taken to avoid any adverse effects on the mechanical properties. From a clinical point of view, the preparation of denture base materials from nanocomposites with low concentrations of NPs is in terms of translucency. However, more research is required to optimize the translucency of 3D-printed resins with and without additives. These improvements can be achieved by modifying the composition of printed resins or by using NPs with a refractive index close to that of the resin.

As a limitation in this study, only one type of NP and one printing orientation were used and the specimen did not replicate the design of a denture. In addition, the heat cycling aging only reflected half a year of intraoral use, and dynamic loading was absent. Moreover, the lack of chromogenic agents and denture disinfectants is considered a significant limitation as these agents have a considerable effect on the color of denture base. Future work should focus on various 3D-printed materials constructed in a denture configuration and subjected to thermal and mechanical stresses similar to those in the intraoral environment, as well as using multiple NP types and concentrations, and printing orientations. In addition, a study of the effect of disinfectants and beverages on the color stability of the introduced nanocomposite is required.

## 5. Conclusions

Compared with the heat-polymerized acrylic resin, both 3D-printed resins showed low translucency values. The translucency was erratically affected by the addition of ZrO_2_NPs. The 3D-printed groups modified with ZrO_2_NPs showed lower translucency than the unmodified groups. All ASIGA samples demonstrated higher translucency than NextDent samples. In terms of low translucency, adding a low concentration of ZrO_2_NPs is more clinically feasible for 3D printable nanocomposite denture base resins.

## Figures and Tables

**Table 1:** Materials and equipment used in the study.

Material Brand Name/Printers/Manufacture/Printing Technology	Composition	Printing Parameters			Post Printing Conditions		
		Layer Thickness	Orientations	Light Source /Wavelength	Rinsing/ Cleaning	Post Curing Machine	Post Curing Time
NextDent Denture 3D+/ NextDent 5100 3D NextDent B.V Soesterberg, The Netherlands/ Stereolithography	Methacrylic oligomers, methacrylate monomer, inorganic filler, phosphine oxides, pigments	50 μm	90°	UV light/405 nm	Isopropyl alcohol 99.9%/ glycerol	LC-3DPrint Box, NextDent, Soesterberg, The Netherlands	10 min
ASIGA DentaBASE ASIGA MAX UV/ASIGA, Erfurt, Germany/Digital light processing (DLP)	7,7,9(or 7,9,9)-trimethyl-4,13-dioxo-3,14-dioxa-5,12-diazahexadecane- 1,16-diyl bismethacrylate; Diphenyl(2,4,6-trimethylbenzoyl) phosphine oxide; Tetrahydrofurfuryl methacrylate	50 μm	90°	UV light/405 nm	Isopropyl alcohol 99.9%/ glycerol	ASIGA Flash, ASIGA, Sydney, Australia	20 min

**Table 2:** Finishing, polishing and thermo-cycling equipment and procedures.

Finishing and Polishing				Thermocycling		
Finishing Paper	Polishing Suspension	Polishing Cloth	Polishing Machine	Machine	Cycles	Temperature/Time
Silicon carbide grinding paper 800, 1500, and 2000 grit	0.050 μm - Master Prep polishing suspension; Buehler GmbH	TexMet C10in, 42-3210; Buehler GmbH, Düsseldorf, Germany	Metaserv 250 grinder-polisher; Buehler GmbH, Lake Bluff, IL, USA	Thermocycler THE-1100/THE-1200, SD Mechatronik GMBH Miesbacher Str. 34 83,620 Feldkirchen-Westerham Germany	5000 cycles	5–55 °C /30 s of dwell time and 5 s for dripping

**Table 3:** Translucency mean values, SD, and significance between HP and NextDent test groups.

Material	ZrO_2_NP Concentration	Mean (SD)	*p*-Value
HP	Control	11.04 (1.3)	0.000 *
NextDent	0%	6.32 ± 0.48 ^a,b,c^	
	0.5%	6.40 ± 0.55 ^a,d,e^	
	1%	6.12 ± 0.33 ^b,d,f^	
	3%	5.66 ± 0.27 ^c,e,f,g^	
	5%	4.91 ± 0.35 ^g^	

* Statistically significant at a level of 0.05. Small letters indicate an insignificant difference between the pairs.

**Table 4:** Translucency mean values, SD, and significance between HP and ASIGA tested groups.

Material	ZrO_2_NP Concentration	Mean (SD)	*p*-Value
HP	Control	11.04 ± 1.3	0.000 *
ASIGA	0%	9.26 ± 0.48 ^a,b^	
	0.5%	8.86 ± 0.75 ^a,c^	
	1%	8.40 ± 0.47 ^b,c^	
	3%	7.13 ± 0.47 ^d^	
	5%	7.46 ± 0.23 ^d^	

* Statistically significant at a level of 0.05. Small letters indicate an insignificant difference between the pairs.

**Table 5:** Pair-wise comparison of concentration levels between the two materials.

		ASIGA				
		0%	0.5%	1%	3%	5%
NextDent	0%	0.000 *	0.000 *	0.000 *	0.005 *	0.000 *
	0.5%	0.000 *	0.000 *	0.000 *	0.02 *	0.000 *
	1%	0.000 *	0.000 *	0.000 *	0.000 *	0.000 *
	3%	0.000 *	0.000 *	0.000 *	0.000 *	0.000 *
	5%	0.000 *	0.000 *	0.000 *	0.000 *	0.000 *

* Statistically significant at a level of 0.05.

**Table 6:** Two-way ANOVA results for the combined effects of materials and concentration levels.

Source	Type III Sum of Squares	df	Mean Square	F-Value	*p*-Value
Corrected Model	329.752a	10	32.975	94.621	0.000 *
Intercept	5839.598	1	5839.598	16,756.418	0.000 *
Material	137.476	1	137.476	394.479	0.000 *
Concentration	42.095	4	10.524	30.198	0.000 *
Material * concentration	5.880	4	1.470	4.218	0.003 *
Error	34.501	99	0.348		
Total	6412.446	110			

* Statistically significant at a level of 0.05.

## Data Availability

Not applicable.
